# Digital connectivity and healthy aging in China: how Internet use shapes subjective well-being through social engagement and isolation

**DOI:** 10.3389/fpubh.2025.1669456

**Published:** 2025-11-20

**Authors:** Jun-Feng Kuang, Lin-Lin Yang, Shang-Zhou Li, Xue-Qing Tian

**Affiliations:** 1 School of Law and Political Science, Yunnan University of Finance and Economics, Kunming, China; 2 Southwest Frontier Minority Research Center, Yunnan University, Kunming, China; 3 School of Medicine, Yunnan University, Kunming, China; 4 College of Humanities and Social Science, Yunnan Agricultural University, Kunming, China

**Keywords:** internet use, subjective well-being, social engagement, social isolation, older adults, China

## Abstract

While the relationship between Internet use and subjective well-being has attracted substantial scholarly attention, empirical evidence regarding its causal mechanisms remains inconclusive. This study examines how Internet use influences subjective well-being among older adults using survey data from 518 respondents across six communities in Kunming, Yunnan Province, China. Results indicate that Internet use demonstrates a significant positive association with subjective well-being when mediating variables are excluded from the analysis. However, the direct effect becomes statistically non-significant after introducing social engagement and social isolation as parallel mediators, suggesting full mediation. These findings reveal that Internet use enhances subjective well-being indirectly through its effects on reducing social isolation and promoting social engagement among older adult populations. Grounded in social support theory, this research provides culturally contextualized insights for developing digital inclusion strategies to promote healthy aging in China.

## 1 Introduction

According to data from the National Bureau of Statistics of China, by the end of 2023, it is estimated that the population of people aged 60 or above will reach 296.97 million, accounting for 21.1% of the total population. It is also estimated that the number of those aged 65 or above will be 216.76 million, representing 15.4% of the total population. With the acceleration of population aging, associated social issues have become increasingly prominent. The complete detachment of older adults from productive society often implies an increased social burden (1). Some studies suggest that Internet use can help older adults overcome psychological or physical barriers and enhance their subjective well-being (2). However, the academic community has not reached a consensus on the relationship between these two variables. One perspective argues that Internet use can improve subjective well-being by promoting healthy behaviors (3), building social capital (4, 5), and fostering social engagement or family relationships (6, 77). In contrast, another viewpoint posits that excessive Internet use is associated with mental health issues in older adults, such as higher levels of depression and anxiety compared to other groups (7), and stronger feelings of loneliness among frequent Internet users (8). Thus, although the impact of Internet use on the subjective well-being of older adults is considerable, the exact pathways of this influence remain unclear. Further studies have attempted to address this issue. For instance, a study on social networks suggests that integrating bonding and bridging social capital can significantly moderate the relationship between Internet use and subjective well-being (4, 5). Another study conducted during the pandemic indicates that Internet use is significantly correlated with loneliness (9), and that loneliness—together with satisfaction with interpersonal relationships—fully mediates the effect of digital media use on personal subjective well-being (10).

Although previous studies have examined the association between Internet use and subjective well-being in older adults, we argue that the mediating roles of social engagement and social isolation in this relationship deserve greater attention. From an individual perspective, ageing is an irreversible process: marked losses in resilience, intrinsic capacity and physical function frequently precipitate psychological distress. As people age, role disruption and status decline often reduce social engagement, intensify loneliness and diminish subjective well-being, thereby accelerating the ageing experience itself (11–13). Consequently, maintaining social engagement is critical. In addition, research has shown that social isolation mediates the link between public-transport use and subjective well-being among older adults (14), and a COVID-19-period study found a significant negative relationship between social isolation and subjective well-being in this population, with loneliness acting as a mediator (15). Taken together, social engagement and social isolation appear to be important mediating variables. Although existing work has established the overall effect of Internet use on subjective well-being (16–18), the possibility that social engagement and social isolation mediate this effect has rarely been examined.

**Figure 1:**
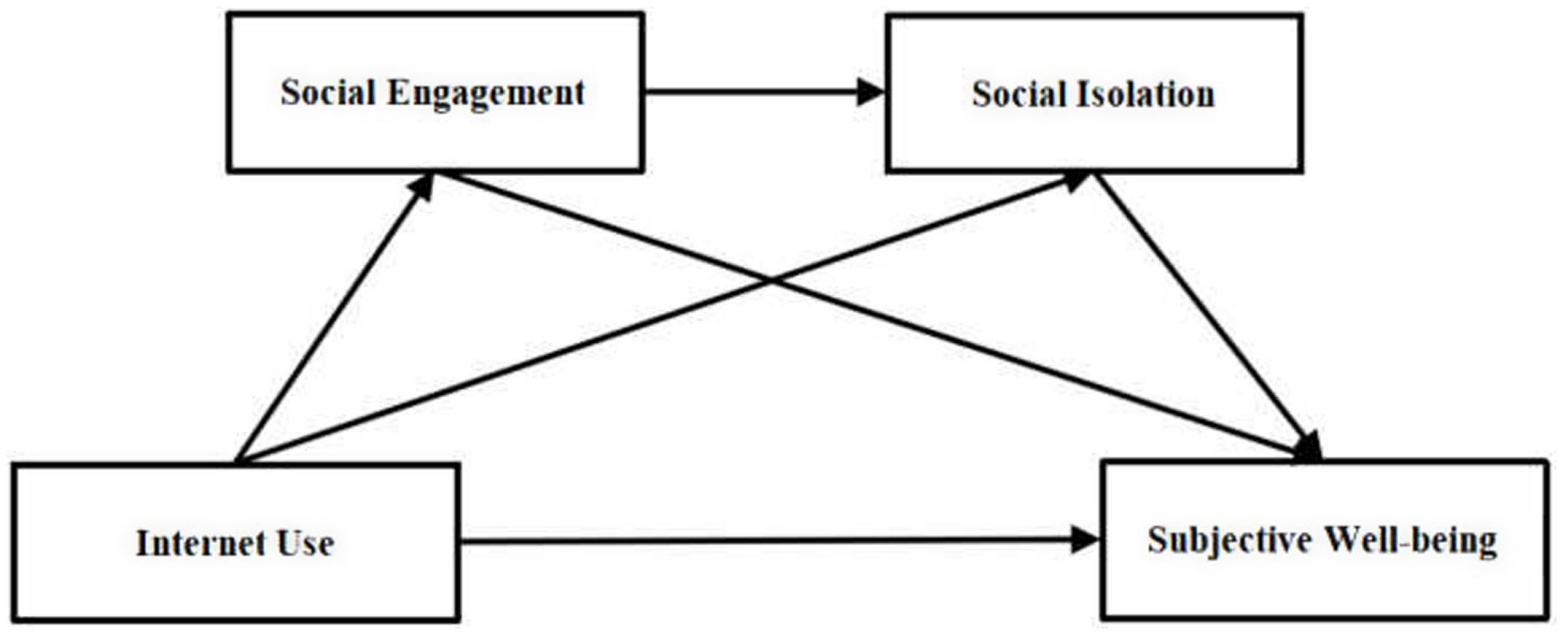
Research model.

Grounded in social support theory, this study examines how Internet use, social engagement and social isolation jointly shape the subjective well-being of older adults in six Kunming (Yunnan Province) communities. By integrating the four constructs into a single model (Figure 1), we clarify the mediating pathways through which social engagement and social isolation link Internet use to subjective well-being. The findings should refine existing theory, offer cross-cultural evidence and illuminate new leverage points for intervention. On the basis of the results, we will propose concrete strategies to enhance older adults’ subjective well-being.

## 2 Literature review and research hypotheses

### 2.1 Internet use and subjective well-being

Subjective well-being (SWB) refers to individuals’ cognitive and affective evaluations of their own lives, ranging from despair and depression to euphoria and satisfaction (19). SWB is usually conceived as a three-dimensional construct: (1) affective experiences of happiness in daily life, (2) positive functioning, meaning or purpose in life, and (3) global life satisfaction (20). For older adults, SWB is typically captured by life satisfaction and mental state, with the persistence of happiness as its core (21). Previous studies have identified a variety of antecedents—children’s self-esteem (22, 23), parenthood (24), exercise (25–27), social-media use (28), parental education and Internet use (29), among others. Internet use is one of the most frequently examined predictors. Media-psychology research has produced two competing findings. One strand shows that Internet use improves SWB by strengthening social ties and providing informational and emotional resources (4, 5, 78). The opposite strand finds that heavy Internet use displaces intimate, kin-based interactions with “weak-tie” contacts, thereby increasing loneliness and depressive symptoms (73, 79).

Social-support theory argues that emotional, informational and material assistance transmitted through network ties is a primary determinant of SWB (30, 31). Internet use is a communication technology that can enlarge, activate and diversify these supportive exchanges: it sustains intergenerational contact, supplies health information and delivers emotional comfort (32, 33). Meta-analytic and longitudinal studies show that the resulting increase in perceived and enacted support raises happiness and life satisfaction among older adults (34–36). Although heavy, compulsive use can crowd out kin ties and lower SWB (73, 79), the dominant evidence indicates that competence-based, moderate Internet use expands supportive resources and enhances SWB.

*Hypothesis 1:* Internet use has a significant positive effect on the subjective well-being of older adults.

### 2.2 The mediating role of social engagement

Social engagement as a communicative-rights concept was first introduced by Barron (37), who argued that audiences should both receive information from, and contribute content to, mass media. Sociologically, social engagement is one of the three pillars of positive-ageing theory (38): older adults actively participate in social and economic life according to their interests and capacities, thereby meeting spiritual needs and realizing self-worth. As online social interaction has blurred the boundaries between the physical and virtual worlds, Internet access has become a new tool for boosting older adults’ social engagement (39–41). Yet academic consensus on the direction of this relationship is still lacking. Empirical results remain mixed. Studies grounded in activity theory and re-socialization perspectives show that Internet use expands social networks and increases engagement levels (4, 5, 42, 43). Conversely, research inspired by the displacement hypothesis finds that excessive online time reduces face-to-face contact and predicts depressive symptoms (44, 45). Li et al. (46), for example, report that heavy Internet use is significantly associated with depression among Chinese older adults. We therefore assume an inverted-U pattern: moderate Internet use facilitates social engagement, whereas Internet addiction undermines it.

Social-support theory construes social engagement as behavioral access to network-based resources: every new role, group or interaction episode enlarges the pool of emotional, informational and appraisal support that directly elevates SWB (30, 31). The Internet functions as a low-cost, high-reach support broker: it removes temporal, spatial and mobility barriers, thereby creating additional role opportunities (online kin contact, interest groups, voluntary associations) and multiplying supportive exchanges (32, 33). Empirical studies show that older adults who acquire supportive ties through Internet-mediated engagement report higher life satisfaction and positive affect (47, 48). While displacement research warns that excessive use may crowd out intimate ties (45), the dominant evidence indicates that competence-based Internet use expands supportive networks, increases engagement and, via the support–well-being link, enhances SWB.

*Hypothesis 2*: Social engagement mediates the relationship between Internet use and the subjective well-being of older adults.

### 2.3 The mediating role of social isolation

Social isolation—also termed social exclusion—is typically defined as a state in which individuals have infrequent or no interaction with the wider society and consequently lack meaningful social relationships (49). Fei’s (50) “differential mode of association” depicts interpersonal ties as concentric circles that weaken as they expand outward, making peripheral ties fragile. Retirement intensifies this process: once work-centred networks dissolve, older adults’ social contacts often contract to the household, heightening loneliness (51). Physical and cognitive decline further curtail mobility and social participation, deepening exclusion (52). Internet-based communication can offset these constraints by removing temporal and spatial barriers, thereby expanding older adults’ social networks and mitigating isolation (53).

Social-support theory treats social isolation as a structural deficit: the fewer and weaker the ties, the lower the flow of emotional, instrumental and informational assistance that buffers stress and sustains subjective well-being (30). Internet use functions as a “support expander.” First, it creates new weak-tie bridges and preserves existing strong ties, restoring emotional and companionship support (54, 55). Second, online health-information seeking supplies instrumental and appraisal support that improves perceived physical condition and life satisfaction, further reducing isolation (43). As isolation diminishes, the supportive exchanges necessary for positive affect and life-evaluation are replenished, lifting SWB (30, 56).

*Hypothesis 3*: Social isolation mediates the relationship between Internet use and the subjective well-being of older adults.

### 2.4 The chain-mediating role of social engagement and social isolation

Social isolation—an objective deficit of meaningful, supportive relationships indexed by network size, composition and interaction frequency (57)—has emerged as a key threat to older adults’ welfare, health and quality of life (58, 59). Conversely, social engagement, one of the three pillars of positive-ageing theory (38), can repair relational deficits: higher engagement is consistently associated with lower isolation (60, 61). Mahmoudi et al. (62) found that 30% of 1,280 Iranian older adults were socially isolated, and that low engagement, unemployment, living alone and low education predicted isolation. Li et al. (22, 23) further showed that diminished engagement erodes community belonging and social capital, thereby reducing quality of life. Engagement and isolation are thus significantly and negatively correlated. A large literature demonstrates that both variables independently predict subjective well-being (63, 64). Participation in art-and-craft groups, for example, gives older adults a sense of purpose and reciprocal appreciation that raises SWB (80). Nakagomi et al. (3) showed that interactions with friends and levels of engagement and isolation jointly shape health and well-being. Foettinger et al. (65) concluded that Internet-based engagement prevents isolation and enhances well-being.

*Hypothesis 4*: Social engagement and social isolation jointly serve a chain-mediating role in the relationship between Internet use and the subjective well-being of older adults.

## 3 Methods

### 3.1 Participants and sample

This study received approval from the Ethics Review Committee of Humanities and Social Sciences at Yunnan Agricultural University and was conducted in full accordance with applicable ethical guidelines and regulations. Prior to data collection, all participants provided informed consent either by signing a written consent form or by verbally confirming their understanding and agreement after the consent document had been read aloud to them.

#### 3.1.1 Participants: inclusion and exclusion criteria

Pursuant to Article 2 of the Law of the People’s Republic of China on the Protection of the Rights and Interests of Older Adults, “older adult” is defined as citizens aged 60 years or older. Consequently, the analytic sample was restricted to individuals who were ≥ 60 years of age at the time of recruitment. Detailed inclusion and exclusion criteria are summarized in Table 1.

**Table 1:** Participants: inclusion and exclusion criteria.

Criteria	
Inclusion criteria	
1	Age ≥ 60 years (in accordance with Article 2 of the Law of the People’s Republic of China on the Protection of the Rights and Interests of Older Adults).
2	Registered or de-facto resident in the selected Kunming communities for at least 6 months.
3	Able to communicate verbally in Mandarin or the local dialect.
Exclusion criteria	
1	Severe cognitive impairment.
2	Critical illness or significant hearing/visual impairment that prevents completion of the interview.
3	Refusal to participate.

#### 3.1.2 Community selection procedure

The sampling frame was the official “Age-Friendly Community” registry published by the Kunming Civil Affairs Bureau. Kunming was first stratified into three concentric zones—core, inner-ring, and outer-ring—defined by population density and per-capita disposable income. Within each zone we randomly selected two sub-districts; in every sampled sub-district we chose the first community that (a) had ≥ 15% residents aged ≥ 60 and (b) provided either a digital activity room or public Wi-Fi. The six selected sites are:

Core zone—Huashan Sub-district and Daguan Sub-district (both Wuhua District);
Inner-ring—Longxiang Sub-district (Wuhua District) and Guandu Sub-district (Guandu District);
Outer-ring—Wujiaying Sub-district (Chenggong New Area) and Jinfang Sub-district (Anning City).

#### 3.1.3 Questionnaire distribution and recovery

Data were collected between September and November 2023 in six Kunming communities. Using a stratified cluster design, 590 self-administered questionnaires were dispatched (98–99 per site); approximately 5% were retained on site as spoilage. After discarding incomplete or logically inconsistent returns, 518 valid questionnaires remained. Respondents’ socioeconomic profiles are reported in Table 2.

**Table 2:** Socio-economic characteristics of the sample (*N* = 518).

Characteristic variable	Category/group	*n*	%
Gender	Male	261	50.4
	Female	257	49.6
Age (Years)	60–70	120	23.3
	71–80	214	41.3
	81–90	138	26.6
	> 90	46	8.9
Education	High school and below	82	15.8
	Junior college	91	17.6
	Bachelor’s degree	310	59.8
	Master’s degree and above	35	6.8
Income	Less than 90,000 yuan	208	40.2
	90,000 yuan to 130,000 yuan	220	42.5
	More than 130,000 yuan	90	17.3

### 3.2 Measures

#### 3.2.1 Internet use

Internet use was operationalized with the 7-item scale developed by James (66). Representative items include “browsing platforms such as Kuaishou” and “sending instant messages.” Respondents rated each item on a 5-point Likert scale (1 = almost never, 5 = very frequently). Cronbach’s *α* for the present sample was 0.936.

#### 3.2.2 Social engagement

Social engagement was measured with the 8-item scale validated by Park and Kim (67). The instrument captures both formal participation (e.g., volunteering) and informal contact (e.g., visiting friends, relatives, or neighbors). Items are rated on a 5-point Likert scale (1 = almost never; 5 = very frequently). Cronbach’s α in the current sample was 0.939.

#### 3.2.3 Social isolation

Social isolation was indexed with the 4-item scale originally developed by Undén and Orth-Gomér (68). Representative items include “When I need help, it is hard to find someone” and “I rarely feel cared for by neighbors or friends.” The wording was slightly adapted to the Chinese cultural context. Each item was rated on a 5-point Likert scale (1 = strongly disagree; 5 = strongly agree). Cronbach’s α for the present sample was 0.968.

#### 3.2.4 Subjective well-being

Subjective well-being was assessed with the WHO-5 Well-Being Index, a brief, psychometrically robust measure developed by the World Health Organization and extensively validated across cultures (69–71). A sample item is “I have felt calm and relaxed.” Responses were recorded on a 5-point Likert scale (1 = strongly disagree; 5 = strongly agree). Cronbach’s *α* in the current sample was 0.942.

### 3.3 Analysis strategy

A two-step analytical strategy was adopted. First, descriptive statistics and zero-order correlations were computed with SPSS 25.0. Second, the SPSS PROCESS macro (Model 4, 5,000 bootstrap samples) was used to estimate the indirect effects of Internet use on older adults’ subjective well-being through social engagement and social isolation. The direct effect of Internet use on subjective well-being was estimated prior to evaluating the hypothesized mediation pathways.

## 4 Results

### 4.1 Descriptive statistics and correlations

Table 3 presents the descriptive statistics, correlation analysis, and reliability test results for all the study variables. The results revealed that Internet use had a significant positive relationship with subjective well-being (*r* = 0.702, *p* < 0.01) and social engagement (*r* = 0.708, *p* < 0.01), while showing a significant negative correlation with social isolation (*r* = −0.781, *p* < 0.01). Additionally, social engagement was positively correlated with subjective well-being (*r* = 0.831, *p* < 0.01), whereas social isolation was negatively correlated with subjective well-being (*r* = −0.711, *p* < 0.01). Additionally, the reliability coefficients (Cronbach’s α) for Internet use, social engagement, social isolation, and subjective well-being ranged from 0.936 to 0.968, all of which exceed the minimum threshold of 0.70. Therefore, the scales used in this study demonstrate high internal consistency hold of 0.70. Therefore, the scales used in this study demonstrate high internal consistency.

**Table 3:** Means, standard deviations and correlation coefficients between variables.

Variable	1	2	3	4	5	6	7	8
1 Gender	1							
2 Age	0.007	1						
3 Education	−0.001	−0.212^**^	1					
4 Income	−0.017	−0.059	0.466^**^	1				
5 Internet use	−0.020	−0.007	0.026	0.106^*^	0.936			
6 Social engagement	−0.012	0.070	−0.147^**^	−0.009	0.708^**^	0.939		
7 Social isolation	0.023	0.070	−0.029	−0.140^**^	−0.781^**^	−0.711^**^	0.968	
8 Subjective well-being	−0.024	0.044	−0.075	0.078	0.702^**^	0.831^**^	−0.764^**^	0.942
Mean	0.500	3.210	2.580	5.620	3.916	3.550	2.297	3.873
SD	0.500	0.900	0.835	2.282	0.824	0.886	0.926	1.199

*N* = 518, ^*^p < 0.05 (2-tailed), ^**^p < 0.01 (2-tailed).

### 4.2 Hypothesis testing

**Figure 2:**
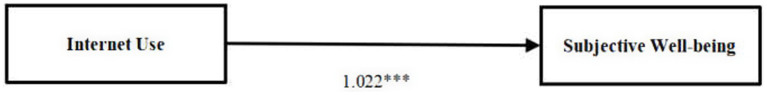
Direct effect of internet use on subjective well-being among older adults.

Hypothesis testing proceeded in two stages. First, prior to entering any mediators, Internet use exerted a significant positive direct effect on older adults’ subjective well-being (*β* = 1.022, *p* < 0.001, R^2^ = 0.493), supporting Hypothesis 1; the corresponding direct-effect model is displayed in Figure 2.

**Figure 3:**
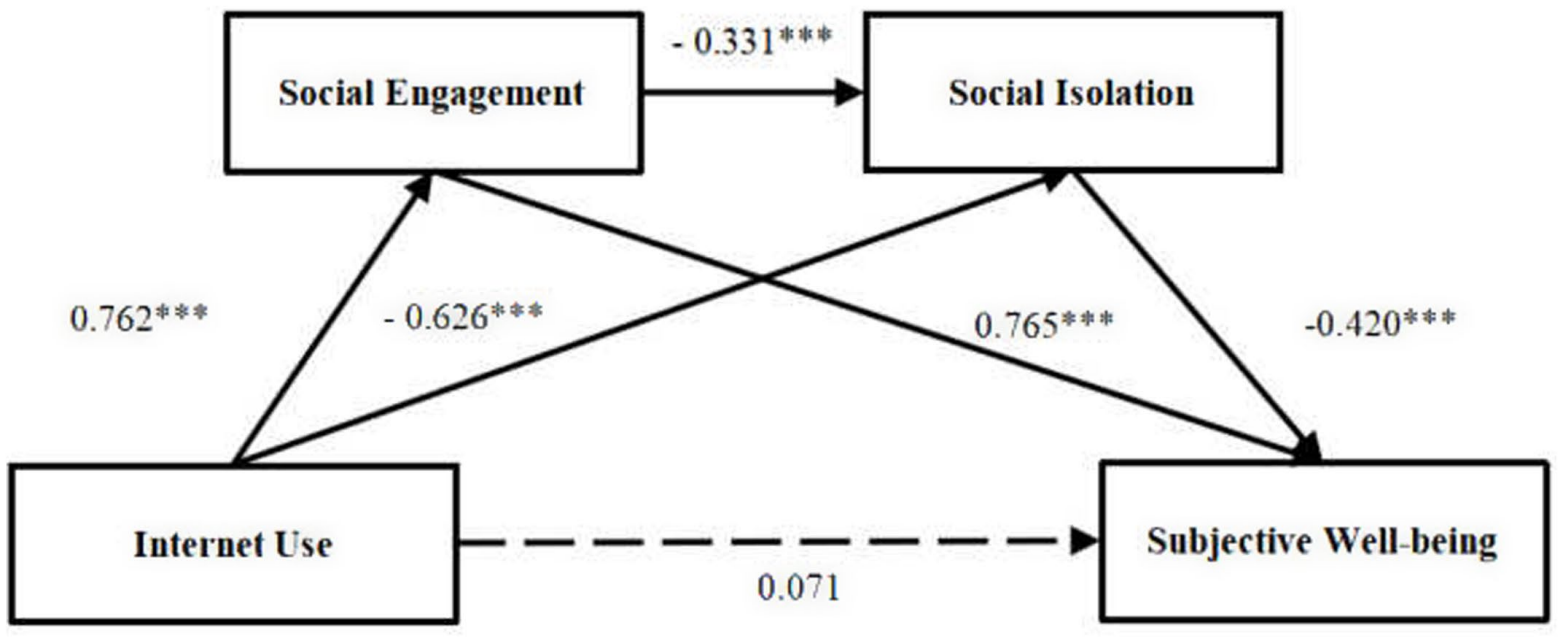
The mediation analysis of social engagement and social isolation between Internet use and subjective well-being.

Second, we introduced the two mediators—social engagement and social isolation—into the model (Table 4). After their inclusion, the direct effect of Internet use on subjective well-being became non-significant (β = 0.071, *p* > 0.05, R^2^ = 0.752), indicating full mediation. Internet use significantly predicted social engagement (β = 0.762, *p* < 0.001, R^2^ = 0.501) and social isolation (β = −0.626, *p* < 0.001, R^2^ = 0.660). Social engagement, in turn, raised subjective well-being (β = 0.765, *p* < 0.001, R^2^ = 0.752) and lowered social isolation (β = −0.331, *p* < 0.001, R^2^ = 0.660), whereas social isolation reduced subjective well-being (β = −0.420, *p* < 0.001, R^2^ = 0.752). Bootstrap estimation (5,000 resamples) yielded a total indirect effect of 0.952 [0.860, 1.044], decomposed into Internet use → social engagement → subjective well-being (0.583 [0.478, 0.699], 61.2%), Internet use → social isolation → subjective well-being (0.263 [0.181, 0.366], 27.6%), and Internet use → social engagement → social isolation → subjective well-being (0.106 [0.064, 0.154], 11.1%); all confidence intervals exclude zero, confirming the chain-mediation model (Figure 3) and supporting Hypotheses 2–4.

**Table 4:** Direct and indirect effects from structural model.

Variable	Direct effect				Indirect effect		
	→M1	→M2	→Y		Estimate	95% CI	
						Lower	Upper
Internet use	0.762^***^	−0.626^***^	0.071	1.022^***^			
	(0.034)	(0.041)	(0.055)	(0.046)			
Social engagement		−0.331^***^	0.765^***^				
		(0.038)	(0.045)				
Social isolation			−0.420^***^				
			(0.049)				
IS→SE → SWB					0.583	0.478	0.699
IS→SI → SWB					0.263	0.181	0.366
IS→SE → SI → SWB					0.106	0.064	0.154
R^2^	0.501	0.660	0.752	0.493			

*N* = 518, ^*^p < 0.05, ^**^p < 0.01, ^***^p < 0.001. IU, Internet Use; SE, Social Engagement; SI, Social Isolation; SWB, Subjective Well-being.

Additionally, because PROCESS estimates OLS paths with bias-corrected bootstrapping and does not provide an overall fit index, we replicated the mediation model in AMOS. The goodness-of-fit statistics (χ^2^/df = 2.07, RMSEA = 0.047, CFI = 0.973, SRMR = 0.035) indicate acceptable model fit and corroborate the PROCESS findings.

## 5 Discussions

The objective of this study is to explore the relationship between Internet use and subjective well-being among older adults, along with the underlying mechanisms that influence this relationship.

Firstly, prior to the inclusion of any mediating constructs, Internet use exerted a significant and positive direct effect on the subjective well-being of Chinese older adults. This finding converges with prior meta-analyses conducted in both Western and East-Asian contexts (32, 33, 35, 36). Social-support theory further contends that the material and emotional resources embedded in digitally expanded networks directly protect mental health (30, 31, 72). Nevertheless, echoing the “moderate-use” caveat repeatedly reported in Western samples (73, 74), we observed a pronounced curvilinear threshold: once daily screen time exceeded approximately 3 h, the marginal gain in social engagement plateaued and subsequently reversed. Within China’s collectivistic culture—where face-to-face rituals remain the gold standard of filial interaction—this inflection point implies that Internet use enhances subjective well-being primarily when it functions as a scheduling tool for offline joint activities rather than as a solitary end in itself.

Second, social engagement and social isolation function as parallel mediators in the association between older adults’ Internet use and subjective well-being. Satici (75) underscored the conceptual complexity of this linkage and called for integrative models that explicitly model intervening psychosocial processes. In line with this recommendation, the present findings reveal that social engagement partially mediates the effect of Internet use on subjective well-being: more frequent Internet use expands opportunities for social engagement, thereby elevating subjective well-being. This result corroborates earlier evidence that digital engagement enhances older adults’ subjective well-being by fostering social integration (36, 76). Conversely, social isolation also emerges as a significant, yet inverse, mediator. Recent studies indicate that Internet use can directly reduce social isolation among older adults (32, 33). Consequently, moderate and purposeful Internet use lowers isolation, which in turn contributes to higher levels of subjective well-being. Taken together, the data suggest that the welfare effects of Internet use in later life are simultaneously channelled through increases in social engagement and decreases in social isolation.

Third, unlike Western samples where Internet use often shows a sizeable direct effect on SWB (32, 35), our Chinese older-adult model yields almost full mediation through social engagement and social isolation. We argue that this divergence is rooted in two intertwined cultural–structural features. First, filial piety (xiao) still prescribes inter-generational co-residence and frequent face-to-face contact (81, 82); thus the “baseline” emotional reward of simply being online is weaker unless it is ritualized into offline visits (e.g., arranging a family dinner via WeChat). Second, structural barriers—hukou-based welfare segmentation and the rural–urban digital infrastructure gap—mean that many older adults must rely on community peer support rather than autonomous, individualized browsing to gain tangible benefits. Once Internet use succeeds in re-embedding seniors into collective activities (square-dancing groups, senior-university Zoom classes), the subsequent reduction in social isolation becomes the principal pathway to SWB, leaving little variance to be explained by a direct effect. In short, the Chinese cultural emphasis on “doing together” converts digital access into well-being only after it is transformed into visible social engagement, thereby producing the near-complete mediation we observe.

## 6 Limitations and directions for future research

This study offers the first Chinese evidence that social engagement and social isolation fully chain-mediate the link between Internet use and subjective well-being; five limitations follow.

First, cross-sectional data: the single-wave design precludes causal inference; future studies should employ two-wave panels or experience-sampling methods to chart intra-individual change. Second, self-selection and self-report biases: older adults willing to complete questionnaires in digitally equipped communities are likely more affluent, healthier, and more motivated to use the Internet; random-digit-dial or registry-based sampling that includes non-users and passive users is needed. Third, culturally salient mediators—intergenerational support, cognitive function, and digital literacy—were omitted; future work should integrate these variables to capture the full Chinese mechanism linking Internet use to late-life well-being. Fourth, the urban, digitally enabled sample: all six communities were located in Kunming and already offered public Wi-Fi or digital activity rooms; older adults in rural areas with poor broadband access or low technological literacy are under-represented. Replicating the model in remote counties and testing whether infrastructural investments or digital-literacy interventions moderate the observed pathways would clarify external validity. Fifth, moderator vacuum: individual differences (personality, vision impairment, chronic conditions) and contextual factors (community public-service density, ethnic minority status) may amplify or dampen the benefits of Internet use; integrating multilevel modeling and propensity-score techniques will help identify for whom and under what conditions Internet use yields the largest well-being dividends.

## 7 Conclusion

Internet use exerts no direct effect on older adults’ subjective well-being; its influence is transmitted entirely through the sequential pathway of social engagement → reduced social isolation. Digital access therefore yields psychological benefits only when it is converted into meaningful social ties.

Three mutually reinforcing policy levers are indicated: (1) age-sensitive design that legally obliges all public-facing digital services to deploy large fonts, high-contrast interfaces and simplified navigation, coupled with tax incentives for firms obtaining national “older-adult-friendly” certification; (2) digital literacy conceived as social policy through internet-training modules embedded in community health centres, libraries and township cultural stations that foreground social functionalities such as video-calling and neighbourhood WeChat groups rather than purely instrumental skills, thereby accelerating engagement gains; and (3) targeted outreach that integrates internet onboarding into routine home-based care for low-use, high-risk older adults—widowed, rural or low-income—and subsidises peer “silver ambassadors” who deliver ongoing technical and social support, ensuring that digitalisation does not become a new axis of exclusion in China’s ageing society.

## Data Availability

The raw data supporting the conclusions of this article will be made available by the authors, without undue reservation.
